# Diagnostic accuracy of unenhanced post-mortem CT and MRI compared to the non-forensic clinical autopsy: a prospective blinded study

**DOI:** 10.1007/s00428-025-04130-5

**Published:** 2025-06-05

**Authors:** Hanno Hoppe, Isabel Arnold, Nicolas Lange–Herr, Jeremias Klaus, Nicole Schwendener, Julia Brünig, Bastian Dislich, Mafalda Trippel, Yara Banz, Wolf-Dieter Zech

**Affiliations:** 1 Institute of Forensic Medicine, University of Bern, Bern, Switzerland; 2 Institute of Tissue Medicine and Pathology, University of Bern, Bern, Switzerland; 3 Department of Diagnostic, Interventional, and Pediatric Radiology, Bern University Hospital, University of Bern, Inselspital Bern, Switzerland; 4 Department of Health Sciences and Medicine, University of Lucerne, Lucerne, Switzerland

**Keywords:** Post-mortem imaging, Post-mortem computed tomography, Post-mortem magnetic resonance imaging, Clinical autopsy, Diagnostic accuracy

## Abstract

The last decades have seen a constant decline in non-forensic clinical autopsy rates worldwide. In this context, post-mortem computed tomography (PMCT) and post-mortem magnetic resonance imaging (PMMR) might offer an alternative to the clinical autopsy. So far, post-mortem imaging is used routinely only in forensic medicine, but it has not yet been implemented into routine clinical pathology casework. This study aimed to assess the diagnostic accuracy of unenhanced PMCT and PMMR for basic pathology groups and specific diagnoses compared to the clinical autopsy. Post-mortem imaging (PMCT and PMMR) was conducted before autopsy on *n* = 120 non-forensic patients deceased in hospitals in a prospective study. Imaging findings were compared to autopsy findings, and sensitivity, specificity, positive predictive value (PPV), and negative predictive value (NPV) were calculated for causes of death and specific pathologic findings. Some of the diagnoses recorded showed high specificity and NPV of over 85% for both PMCT and PMMR. However, even the combined use of PMCT and PMMR could not visualize various relevant autopsy findings such as those related to sepsis, hematologic malignancies, chronic liver congestion, endomyocardial, myocardial, and liver fibrosis, acute tubular necrosis, aortic valve stenosis, duodenal ulceration, and small macroscopic findings in general. For specific findings, post-mortem imaging showed no significant differences to autopsy and high diagnostic accuracy with over 85% sensitivity. Examples for such findings included the diagnosis of acute myocardial infarction and pulmonary embolism in PMMR, pneumonia in PMCT, as well as pancreatitis, abscesses, metastases, and aortic dissection in both PMCT and PMMR.

**Supplementary information:**

The online version contains supplementary material available at 10.1007/s00428-025-04130-5.

## Introduction

In the last decades, there has been a constant decline in non-forensic clinical autopsy rates worldwide [1–4]. Against the background of clinical autopsy demise, medical experts and scientific literature stress the importance of the clinical autopsy for the medical system. Despite advancements in modern medicine, the clinical autopsy remains an invaluable tool for medical education, quality control of clinical diagnoses, treatment transparency, and the establishment of national cause-of-death statistics [5–8]. Studies demonstrated discrepancies between clinical diagnoses and clinical autopsy findings above 40%. Errors in main clinical diagnoses, which may have influenced therapeutic strategies or even survival of patients, are estimated to occur in 10–25% of clinical cases [9–11]. These numbers stress a critical evaluation of modern diagnostic methods by collection of relevant post-mortem information. The reasons for declining clinical autopsy rates are manifold. On the one hand, there are socio-economic aspects such as restrictive legal regulations demanding approval from the next of kin, religious objections, and negative public perception of “cutting open and eviscerating” a deceased body. On the other hand, the decision for an autopsy is significantly dependent on the motivation of the last treating clinical physician, who is already burdened with delivering the death message to the next of kin. Thus, physicians often avoid further inconvenience by asking for consent to perform a clinical autopsy [1, 3, 12].

However, repetitive statements on the importance of the clinical autopsy in medical publications and public press have not influenced declining autopsy rates [1–4, 12]. Therefore, new approaches and methods need to be evaluated to counterbalance the general loss of relevant post-mortem information due to this decline. In this context, post-mortem imaging may offer a means to counterbalance this development [13, 14]. The introduction of imaging techniques such as post-mortem computed tomography (PMCT) and post-mortem magnetic resonance imaging (PMMR) has opened new diagnostic possibilities in post-mortem examinations [15, 16]. Performed separately, each of the aforementioned imaging techniques has diagnostic shortcomings. While PMCT is particularly apt for the detection of bone pathology and lung findings, it is rather insufficient for the detection of soft tissue and parenchymal organ pathology [17, 18]. PMMR is particularly useful for the judgment of soft tissue in general and parenchymal organs in particular, except the lungs [16, 19, 20].

Currently, post-mortem imaging is routinely utilized only in the field of forensic medicine, where it has been established as a valuable adjunct to forensic autopsy [16, 18, 21]. Post-mortem imaging has not been implemented into routine clinical pathology casework yet. In forensic cases, post-mortem imaging focuses on determining the cause of death and forensic-relevant findings such as traumatic injuries or injury-related gas distributions. However, the non-forensic field of clinical pathology addresses different questions, in particular validation of clinical findings and diagnoses. Moreover, some relevant pathology findings, such as neoplasms, are rather rare or incidental findings at forensic autopsies [5, 8, 13, 14].

In non-forensic autopsy cases, the diagnostic accuracy of PMCT and PMMR has mainly been investigated in fetuses and infants [22–24]. Although there are numerous studies on post-mortem imaging in adults, only few studies investigated its actual diagnostic accuracy. Most of these studies comprised forensic cases and mainly focused on the determination of cause of death, and mostly only one technique, either PMCT or PMMR, was used [14, 15, 20, 21, 25–28]. Larger prospective studies that evaluated the feasibility of unenhanced in-hospital PMCT and PMMR with a focus on adult pathology are scarce. Such studies showed that PMCT and PMMR have the potential for significant diagnostic power compared to the clinical autopsy [13, 28–30]. To date, knowledge of the diagnostic accuracy of PMCT and PMMR compared to the clinical autopsy is available mainly for causes of death. Pathology groups and specific diagnoses were investigated previously, but diagnostic accuracy calculations were often impeded due to the small case numbers of specific findings [13, 28–30]. Therefore, this study aimed to assess the diagnostic accuracy of unenhanced PMCT and PMMR for basic pathology groups and specific diagnoses compared to the clinical autopsy.

## Methods

### Study design and study subjects

The study design was prospective. Between 2018 and 2023, post-mortem imaging (PMCT and PMMR) was conducted on deceased non-forensic patients who died in a hospital and the treating physicians requested clinical autopsy. The hospital included a university hospital and 12 regional hospitals located in the cantons of Bern, Fribourg, and Solothurn in Switzerland. Treating physicians provided information on the patient’s clinical medical history. The next of kin were asked for their permission for autopsy as well as post-mortem imaging. The local ethics committee of the canton of Bern (KEK) approved the study (KEK ID-No. 2017–01201). Inclusion criteria for study subjects were death in a hospital setting and age over 18 years. Exclusion criteria were non-hospital death and age under 18 years.

### Post-mortem imaging

Post-mortem imaging (PMCT and PMMR) was conducted at the Institute of Forensic Medicine Bernprior to clinical autopsy. The time between death and post-mortem imaging ranged from five to 60 hours. After death, the bodies were kept in cold storage until shortly before imaging. Bodies were scanned in the supine position in both PMCT and PMMR. Whole-body PMCT (Siemens Somatom® X.cite) was conducted before PMMR. The PMCT scan protocol was based on the current standards in PMCT [16–18, 31, 32] imaging with the following parameters: tube voltage 140 kV, tube current 700 mAs, slice thickness 1 mm, increment 0.7 mm, field of view (FoV) was adapted to body part width, used filters: bone (Br 60), soft tissue (Br 40), lung (BL60 s). Separate head and neck scans were conducted with the following parameters: 140 kV, 700 mAs, slice thickness 0.8 mm, increment 0.5 mm, FoV was adapted to head width, used filters: bone (Hr 60), soft tissue (Hr 40).

PMMR (3 Tesla, Philips) was conducted immediately after PMCT and corresponded to the current standards of PMMR soft tissue and organ diagnostics [19, 20, 33–36]. The following PMMR protocols and sequences were applied: head axial T1-weighted sequence: relaxation time (TR): 600 ms, echo time (TE): 10 ms, slice thickness: 4 mm, gap: 1 mm, FoV: 230. Head axial T2-weighted sequence: TR: 3000 ms, TE: 80 ms, slice thickness: 4 mm, gap: 1 mm, FoV: 230. Head, neck, and trunc coronary T1-weighted turbo spin echo (TSE) sequence: TR: 614 ms, TE: 8 ms, slice thickness: 5 mm, gap: 0.5 mm, FoV: 530. Head, neck, and trunc coronary T2-weighted DIXON (IP/OP7water/fat reconstructed) sequence: TR: 4077 ms, TE: 80 ms, slice thickness: 5 mm, gap: 0.5 mm, FOV: 530. Head, neck, and trunc axial T1-weighted TSE sequence: TR: 700 ms, TE: 9 ms, slice thickness: 3 mm, gap: 1 mm, FoV: 450. Head, neck, and trunc axial T2-weighted DIXON (IP/OP7water/fat reconstructed) sequence: TR: 6431 ms, TE: 90 ms, slice thickness: 3 mm, gap: 1 mm, FoV: 450. Heart short-axis T2-weighted DIXON (IP/OP7water/fat reconstructed) sequence: TR: 3346 ms, TE: 90 ms, slice thickness: 3 mm, gap: 0.3 mm, FoV: 310. Heart axial T2-weighted 3D sequence: TR: 1500 ms, TE: 248 ms, slice thickness: 0.7 mm, gap: 0.7 mm, FoV: 180. PMMR lasted approximately 3 hours.

### Autopsy

The time between post-mortem imaging and autopsy ranged between 1 and 3 hours. Autopsies were performed at the Institute of Tissue Medicine and Pathology Bern by board-certified clinical pathologists and according to the code of practice for medical clinical autopsies [37]. Autopsies were conducted with knowledge of clinical case information but blinded to post-mortem imaging results. Standard histologic examinations were conducted in each autopsy case. In some cases, further examinations such as Immunohistochemistry were conducted as deemed necessary. All macroscopic and microscopic autopsy findings, as well as the cause of death, were presented in a standardized autopsy report.

### PMCT and PMMR image analysis

PMCT and PMMR image analysis was conducted in a PACS (Sectra Workstation IDS7, Version 20.2.8.3353, 2018, Sectra AB, Linköping) by three observers (a board-certified radiologist with 5 years of experience in post-mortem imaging; a board-certified radiologist with 10 years of experience in post-mortem imaging; a board-certified forensic pathologist with 15 years of experience in post-mortem imaging) independently and blinded to autopsy results. In cases of disagreement of imaging findings, a consensus was reached together in subsequent readings.

### Data analysis and statistics

**Fig. 1:**
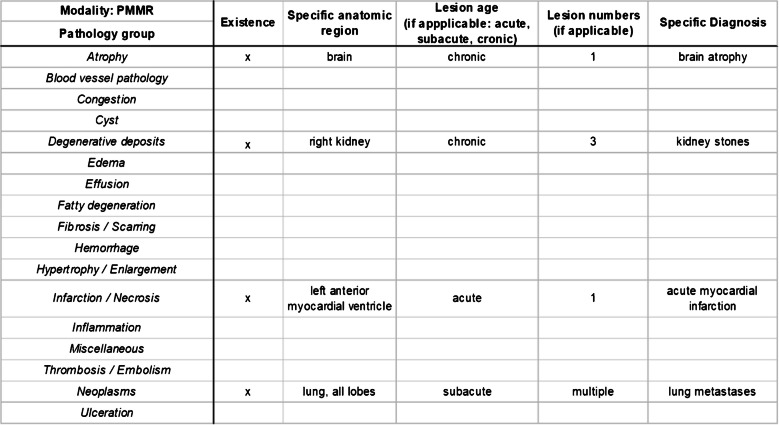
Example of a chart that was used to document pathologic findings. In each single case, such a chart was completed separately for autopsy, PMCT, and PMMR findings. The separate charts were then compared to one another. Findings were assigned to a basic pathology group, the according anatomic region, a lesion age (if applicable), the number of lesions (if applicable), and a definite specific diagnosis. Exemplarily, the presented chart shows the documentation of all PMMR findings in a single case. In particular, PMMR detected brain atrophy, kidney stones, acute myocardial infarction, and lung metastases

Charts were developed for the study that allowed filling in different pathology groups and according specific diagnoses (Fig. 1) as well as causes of death for each case. Pathology groups were defined as groups with basic pathological changes such as infarction or hemorrhage. If these changes were detected in specific organs or tissues (e.g., myocardial infarction or brain hemorrhage), they were defined as specific diagnoses and subsumed under the according pathology group (e.g., the specific diagnosis of myocardial infarction was subsumed under the infarction pathology group).

For each investigated patient, a chart was completed for autopsy, PMCT, PMMR, and PMCT + PMMR findings separately and blinded to each other. The charts were then compared to one another.

SPSS (IBM, Version 23.0) was used for statistic calculations. Fisher’s exact test (for groups with at least *n* = 5 cases and less than 20 cases) or chi-squared test (for groups with at least 20 cases) was used to test whether the diagnoses made were dependent on the examination modality (autopsy, PMCT, and PMMR). The tests were conducted for autopsy vs. PMCT, autopsy vs. PMMR, and autopsy vs. PMCT + PMMR. *P*-values ​​ < 0.05 were assumed as statistically significant. Moreover, sensitivity, specificity, positive predictive values (PPV), and negative predictive values (NPV) were calculated for causes of death, pathology groups, and specific diagnoses. These calculations were done for autopsy vs. PMCT, autopsy vs. PMMR, and autopsy vs. a combined assessment of PMCT and PMMR, each with autopsy as gold standard.

Fleiss’ kappa values were calculated for PMCT and PMMR specific diagnoses initially made by the three observers to determine the interobserver variability. Interobserver variability was defined as the difference in specific diagnoses made between the observers.

## Results

A total of *n* = 120 deceased in-hospital patients (*n* = 75 males with a mean age of 65.7 years and 45 females with a mean age of 71.1 years) were included in the study. The patients had been treated in the following hospital units prior to death: intensive care (*n* = 44), internal medicine (*n* = 42), emergency room (*n* = 6), general surgery (*n* = 8), cardiology (*n* = 5), urology (*n* = 6), gynecology (*n* = 3), nephrology (*n* = 4), and oncology (*n* = 4).

In post-mortem imaging, the kappa values (0.79 PMCT and 0.71 PMMR) showed substantial agreement between the three observers for all specific diagnoses made.

### Causes of death

Table 1 shows the numbers and comparisons for the different causes of death assessed at autopsy, PMCT, and PMMR. The detailed results for the calculations of sensitivity and specificity regarding the comparison of autopsy and imaging are shown in the Supplementary Table 1. The most frequently recorded causes of death at autopsy were pneumonia, cardiac arrest due to existing myocardial disease, coronary thrombosis/myocardial infarction, internal hemorrhage, and neoplasms. In 5% of the cases, the cause of death remained unclear.

**Table 1:** Numbers of causes of death assessed at autopsy, PMCT, and PMMR in alphabetical order. Autopsy was tested against PMCT, PMMR, and PMCT + PMMR using Fisher’s exact test or chi-squared test (* *p* > 0.05, ** *p* < 0.05, and *** *p* < 0.01). No tests were conducted for groups with less than 5 cases or when the imaging numbers were zero

Cause of death	Autopsy	PMCT	PMMR	PMCT + PMMR
Cerebral hemorrhage	6	6*	6*	6*
Cerebral inflammation	1	0	1	1
Cerebral infarction	1	1	1	1
Coronary thrombosis and/or myocardial infarction	12	0	11*	11*
Myocarditis	2	0	1	1
Pericardial tamponade	5	5*	5*	5*
Existing/underlying myocardial disease	12	0	7**	9*
Pulmonary embolism	10	0	8*	8*
Pneumonia	23	19*	14**	22*
ARDS/lung failure	4	3	2	3
Multi organ failure	2	0	0	0
Acute pancreatitis	2	2	2	2
Sepsis	9	0	0	0
Neoplasms	11	8*	9*	9*
Ileus	3	3	2	3
Internal hemorrhage due to dissection/aneurysm	11	11*	11*	11*
Unclear/undetermined	6	5*	5*	5*
**Total**	**120**	62***	84***	96***

For some causes of death (cerebral inflammation, cerebral infarction, myocarditis, ARDS, multi-organ failure, acute pancreatitis, ileus), statistical calculations were not performed due to low case numbers.

For cerebral hemorrhage, pericardial tamponade, internal hemorrhage, and neoplasms, no significant differences between autopsy, PMCT, and PMMR were observed, with 100% sensitivity and specificity for both imaging methods.

PMCT was not implementable for causes of death related to coronary thrombosis and/or myocardial infarction, pre-existing myocardial damage, pulmonary embolism, and sepsis (zero detectability, 0% sensitivity, and 0% PPVs each). Contrary to PMCT, diagnosis of the aforementioned causes of death did not significantly differ between autopsy and PMMR (showing medium to high sensitivities), except for sepsis (zero detectability and 0% sensitivity and PPV, respectively).

The overall sensitivity for the correct determination of the cause of death using post-mortem imaging was 54% for PMCT and 75% for PMMR. A combined use of PMCT and PMMR resulted in an overall higher sensitivity of 85%. For all causes of death, specificities and negative predictive values ranged between 92 and 100% for both PMCT and PMMR.

### Pathology groups and specific diagnoses

**Fig. 2:**
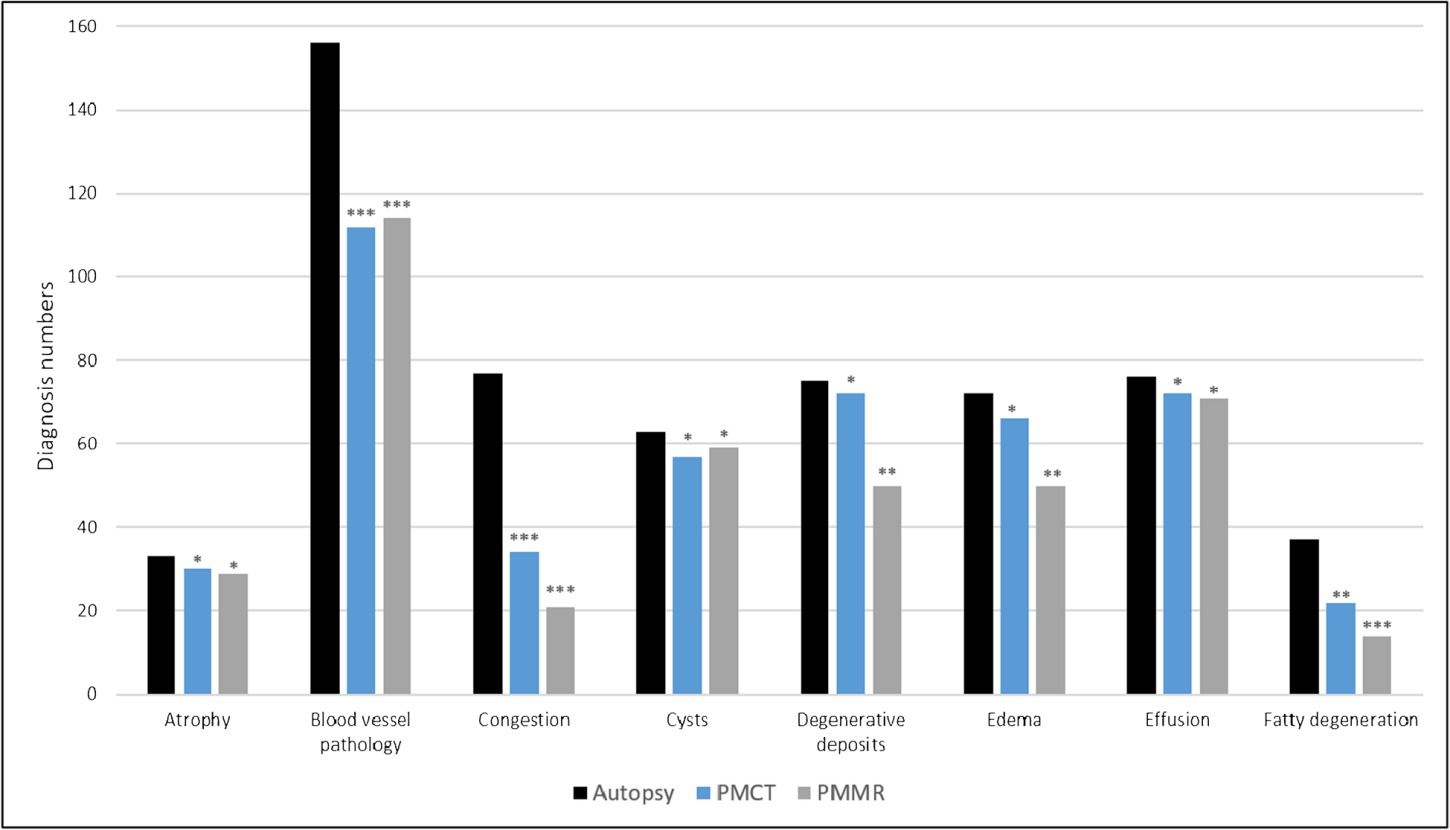
Results of autopsy, PMCT, and PMMR for the detection of different pathology groups (in alphabetical order a–f). Autopsy was tested against PMCT, PMMR, and PMCT + PMMR using Fisher’s exact test or chi-squared test (* *p* > 0.05, ** *p* < 0.05, and *** *p* < 0.01).

**Fig. 3:**
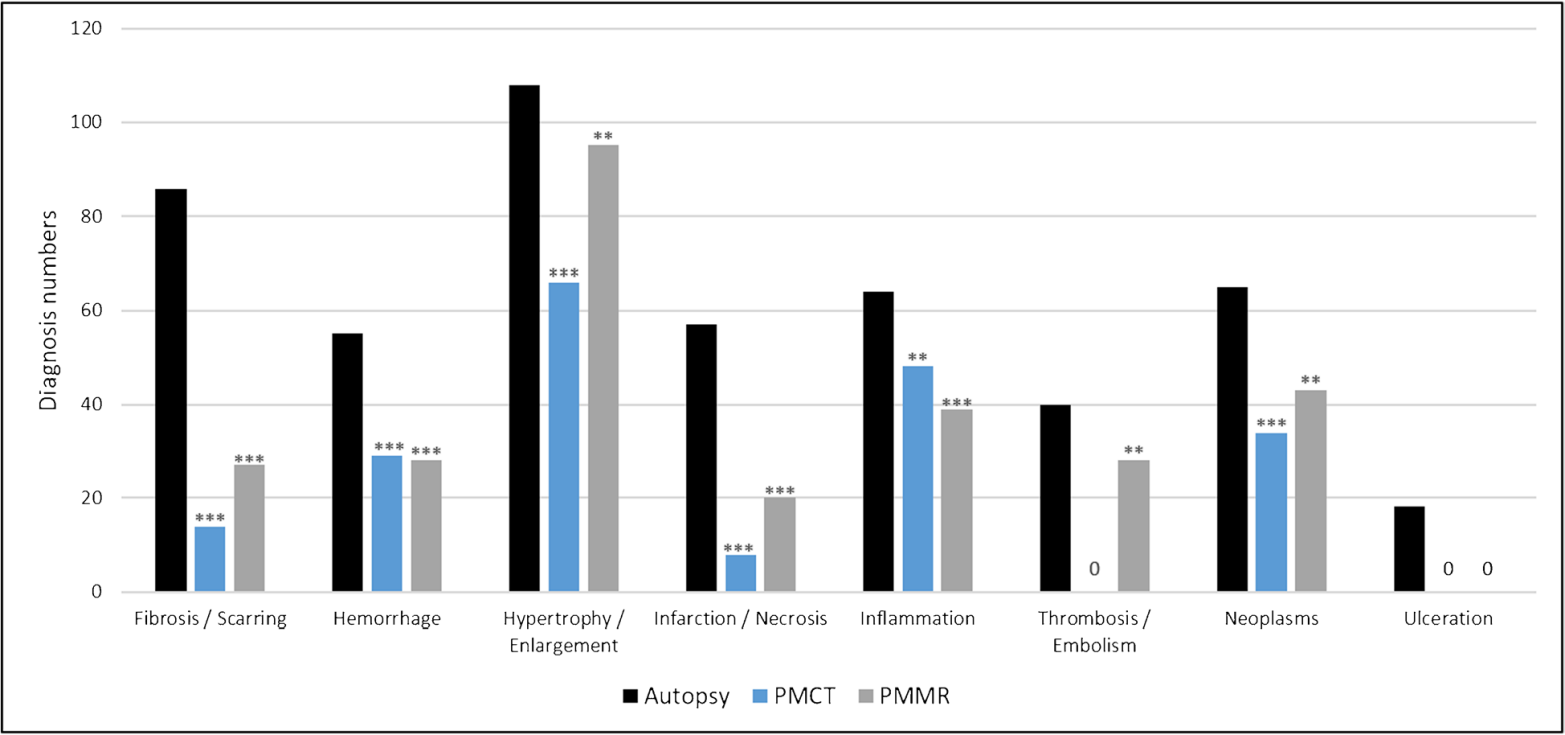
Results of autopsy, PMCT, and PMMR for the detection of different pathology groups (in alphabetical order f–u). Autopsy was tested against PMCT, PMMR, and PMCT + PMMR using Fisher’s exact test or chi-squared test (* *p* > 0.05, ** *p* < 0.05, and *** *p* < 0.01).

Figures 2 and 3 shows the pathology groups recorded at autopsy, PMCT, and PMMR. Tables 2 and 3 shows the numbers and comparison results for all specific diagnoses that were subsumed under each pathology group for autopsy and imaging. A miscellaneous pathology group was formed for different specific pathologies (including aortic valve stenosis, ileus, lymph node increase, pulmonary emphysema, and sigma diverticulosis) that did not fit in any of the other groups. The detailed results for the calculations of sensitivity and specificity for each pathology group and each specific diagnosis can be found in alphabetical order in the Supplementary Tables 2, 3, and 4.

**Fig. 4:**
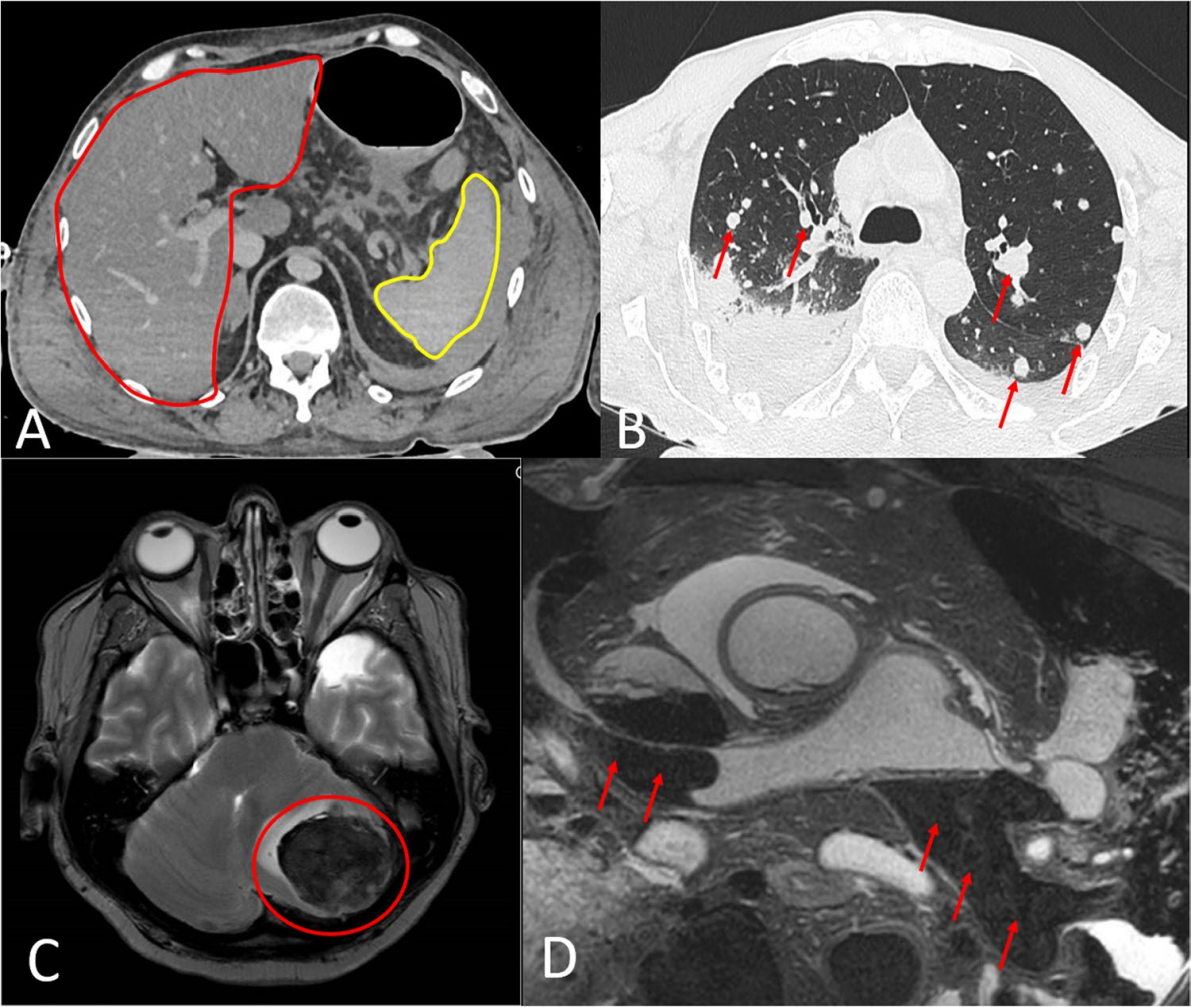
Exemplary PMCT **A**, **B** and PMMR **C**, **D** images of recorded pathologies. **A** Severe liver (red borderline) steatosis, which can be diagnosed due to its hypodense parenchymal appearance compared to the spleen (yellow borderline) parenchyma in PMCT. **B** Multiple lung metastases (red arrows) of unknown origin in PMCT. **C** Malignant neoplasm (red circle) in the left cerebellar hemisphere in PMMR. **D** Pulmonary artery embolism (red arrows) in PMMR

The only pathology groups for which no significant diagnostic differences were found between autopsy, PMCT, and PMMR were atrophy, cysts, and effusions (Figs. 2 and 3). No significant differences between autopsy and imaging were observed for all specific diagnoses subsumed within these pathology groups (Tables 2 and 3). For all other pathology groups, significant differences compared to autopsy were found for at least one of the two imaging modalities. Regarding the specific diagnoses in these pathology groups, both PMCT and PMMR showed no significant difference (*p* > 0.05) to autopsy with medium to high sensitivities and PPVs for abscesses, brain edema, aortic dissection, aortic aneurysm, liver cirrhosis, fatty pancreas, gall bladder stones, large hemorrhages, hypertrophy (except myocardium), ileus, lymph node increase, sigma diverticulosis, and metastases (Fig. 4). Both PMCT and PMMR showed no significant difference compared to autopsy for aortic dissection and aortic aneurysm. In cases of aortic wall rupture due to dissection or aneurysm, neither PMCT nor PMMR were capable of detecting the actual focal rupture site.

**Table 2:** Numbers and comparison of the specific diagnoses that were subsumed under each pathology group (in alphabetical order from a to f) for autopsy and imaging. Autopsy was tested against PMCT, PMMR, and PMCT + PMMR using Fisher’s exact test or chi-squared test (* *p* > 0.05, ** *p* < 0.05, *** *p* < 0.01)**.**

Diagnosis	Autopsy	PMCT	PMMR	PMCT + PMMR
***Atrophy***				
Brain atrophy	9	9*	9*	9*
Kidney atrophy	12	11*	11*	11*
Osteoporosis	12	10*	9*	11*
***Blood vessel pathology***				
Pulmonary artery sclerosis	13	2***	2***	2***
Aortal arteriosclerosis	61	44**	42**	44**
Coronary arteriosclerosis	51	38**	46*	46*
Coronary stenosis	44	0	33**	33**
Aortic dissection all (ruptured and not ruptured wall)	15	13*	14*	14*
Rupture site of aortic dissection	7	1***	3**	3**
Aortic aneurysm all (ruptured and not ruptured wall)	16	15*	15*	15*
Rupture site of aortic aneurysm	7	1***	3**	3**
***Congestion***				
Acute pulmonary congestion	29	25*	13**	25*
Acute liver congestion	21	9***	8***	9***
Chronic liver congestion	12	0	0	0
Acute spleen congestion	15	0	0	0
***Cysts***				
Kidney cyst	31	30*	30*	31*
Liver cyst	11	9*	9*	9*
Ovarial cyst	12	10*	11*	11*
Thyroid cyst	9	7*	8*	8*
***Degenerative deposits***				
Heart valve calcifications	34	32*	16***	32*
Pancreatic calcifications	12	11*	7**	11*
Gall bladder stones	29	29*	27*	29*
***Edema***				
Brain edema	34	33*	33*	33*
Pulmonary edema	38	33*	17**	33*
***Effusion***				
Pleural effusion	43	42*	39*	42*
Pericardial effusion	16	15*	15*	16*
Ascites	17	15*	17*	17*
***Fatty degeneration***				
Fatty liver	27	13**	5***	14**
Fatty pancreas	10	9*	9*	9*
***Fibrosis/scarring***				
Endomyocardial fibrosis	12	0	0	0
Myocardial fibrosis	20	0	0	0
Myocardial scarring	17	0	13*	13*
Pulmonary fibrosis	8	6*	1**	6*
Liver fibrosis	13	0	0	0
Liver cirrhosis	8	8*	7*	8*
Kidney scarring	8	0	6*	6*

**Table 3:** Numbers and comparison of the specific diagnoses that were subsumed under each pathology group (in alphabetical order from h-u) for autopsy and imaging. Autopsy was tested against PMCT, PMMR, and PMCT+PMMR using Fisher's exact test or chi-squared test (* *p* > 0.05, ** *p* < 0.05, *** *p* < 0.01)**.**

Diagnosis	Autopsy	PMCT	PMMR	PMCT + PMMR
***Hemorrhage***				
All hemorrhages except mucosal and endocardial hemorrhage	32	29*	28*	29*
Mucosal and endocardial hemorrhage only	23	0	0	0
***Hypertrophy/enlargement***				
Myocardial hypertrophy	51	10***	39**	39**
Thyroid enlargement	10	10*	10*	10*
Splenomegaly	12	12*	12*	12*
Prostatic hyperplasia	15	14*	14*	14*
Enlarged lymph nodes	20	20*	20*	20*
***Infarction/necrosis***				
Brain infarction	10	8*	8*	8*
Myocardial infarction	13	0	12*	12*
Acute tubular necrosis	34	0	0	0
***Inflammation***				
Abscess	9	8*	9*	9*
Pericarditis	10	0	1***	1***
Pneumonia	32	29*	18**	29*
Pancreatitis	12	10*	10*	10*
***Miscellaneous***				
Aortic valve stenosis	11	0	0	0
Ileus	9	9*	9*	9*
Lymph node increase	21	20*	20*	20*
Pulmonary emphysema	22	18*	8**	20*
Sigma diverticulosis	21	21*	21*	21*
***Thrombosis/embolism***				
Coronary thrombosis	10	0	8*	8*
Pulmonary embolism all	19	0	10**	10**
Central and paracentral pulmonary embolism only	11	0	10*	10*
***Neoplasms***				
Benign neoplasms	23	13**	17*	17*
Malignant primary neoplasms	21	11***	15**	15**
Metastases	12	10*	11*	11*
Hematologic malignancies	9	0	0	0
***Ulceration***				
Gastric ulceration	9	0	0	0
Duodenal ulceration	9	0	0	0

Autopsy detected significantly more primary malignant neoplasms than PMCT and PMMR. PMMR (but not PMCT) showed no significant difference compared to autopsy for benign neoplasms. PMMR exhibited a more than 20% greater sensitivity than PMCT for the diagnosis of both benign and malignant neoplasms. Specific diagnoses in which PMCT (but not PMMR) showed no significant differences (*p* > 0.05) compared to autopsy included pulmonary emphysema and pneumonia. Specific diagnoses that were potentially detectable in some cases of both PMCT and PMMR but showed significant differences (*p* < 0.05) compared to autopsy with medium to low sensitivities and PPVs included arteriosclerosis, acute liver congestion, fatty liver, and myocardial hypertrophy.

Both PMCT and PMMR were not capable of detecting diagnoses such as chronic liver congestion, acute spleen congestion, endomyocardial fibrosis, myocardial fibrosis, liver fibrosis, acute tubular necrosis, aortic valve stenosis, mucosal and endocardial hemorrhage, hematological malignancies, and gastric and duodenal ulcerations.

PMCT-only showed zero detectability for the diagnoses of pericarditis, coronary stenosis, coronary thrombosis, myocardial infarction, myocardial scarring, kidney scarring, and pulmonary embolism. PMMR showed no significant differences compared to autopsy with medium to high sensitivities and PPVs for the aforementioned diagnoses, except for pericarditis (*p* < 0.01, 13% sensitivity).

With a few exceptions, most of the pathology groups and specific diagnoses recorded showed high specificity and high NPV of over 85% for both PMCT and PMMR. Overall, the combined use of PMCT and PMMR did not result in an increase in specificity or NPV greater than 5%.

The combined use of PMCT and PMMR compared to the isolated use of either of the techniques resulted in a moderate increase of sensitivity (10% at maximum) for osteoporosis, atherosclerosis, cysts, pericardial effusion, pulmonary emphysema, and all recorded fatty degenerations. Sensitivity increases greater than 10% for a combined use of PMCT and PMMR were not observed overall.

## Discussion

The results of the present study show that both unenhanced PMCT and PMMR are capable of diagnosing some relevant autopsy findings with relatively high sensitivity and comparable accuracy. However, some relevant findings, such as coronary stenosis or thrombosis, myocardial infarction, and pulmonary embolism could only be diagnosed with PMMR. Various relevant findings, such as sepsis, aortic valve stenosis, or myocardial fibrosis, could not be diagnosed with either PMMR or PMCT. Although the present study did not involve forensic cases, there was an overlap of diagnostic results with the results of previous forensic post-mortem imaging studies [14, 15, 20, 21, 25, 27]. This is likely due to a certain proportion of natural deaths that resemble in-hospital death findings in the forensic setting.

The findings that could easily be diagnosed with either PMCT or PMMR included atrophy, pericardial tamponade, cysts, brain edema, effusions, larger hemorrhages, brain infarction, pancreatitis, abscess, and diverticulosis. PMCT was found to be more sensitive than PMMR for the diagnosis of osteoporosis, calcifications, and pulmonary pathologies such as pulmonary edema, fibrosis, and pneumonia. In line with previous studies, PMMR was highly superior for the diagnosis of most heart-related pathologies. This included the diagnosis of coronary thrombosis, myocardial infarction, and central and paracentral pulmonary embolism although none of the aforementioned diagnoses showed a 100% sensitivity [20, 34–36].

Aortic dissection and aortic aneurysms showed high diagnostic sensitivities in both PMCT and PMMR, which is in line with previous unenhanced forensic PMCT and PMMR studies [14, 20]. However, the localization of the actual focal rupture of an aneurysm or dissection is also of importance in clinical pathology. While the focal rupture could easily be visualized with PMCT angiography, the results of the present study show that this is not possible with either unenhanced PMCT or PMMR and thus limits the diagnostic possibility of unenhanced post-mortem imaging for these particular findings.

In the clinical autopsy, identification and confirmation of neoplasms are generally of importance. To date, there are no studies in which the diagnostic accuracy of unenhanced post-mortem imaging for neoplasms has been systematically investigated in larger numbers. This is likely because post-mortem imaging studies were mostly conducted on forensic cases, in which neoplasms are rather rare and incidental findings. Studies that examined in-hospital clinical autopsy cases only mentioned the presence of neoplasms but did not perform calculations on diagnostic accuracy due to small numbers [13, 14, 20, 28–30]. In the present study, *n* = 65 neoplasms were identified at autopsy. PMMR demonstrated higher sensitivity than PMCT for the diagnosis of both benign and malignant neoplasms. However, with the exception of metastases PMMR only showed moderate sensitivity overall. Nevertheless, based on the results, PMMR should be preferred to PMCT for the diagnosis of neoplasms. In the present study, it was noticed that particularly very small findings were difficult to diagnose with both PMCT and PMMR. This was mainly the case with neoplasms smaller than 0.5 cm and it may partly explain the relatively low sensitivity for detection of neoplasms overall. Both PMCT and PMMR were completely unsuitable for diagnosing hematological malignancies, which further reduced the diagnostic accuracy for malignant neoplasms overall.

The study results showed that both PMCT and PMMR could not be used for the diagnosis of intestinal mucosal ulcerations or hemorrhages, endomyocardial or myocardial fibrosis, liver fibrosis, acute tubular necrosis, and pericarditis. Some of these findings can be of great relevance depending on the case constellation but may only be verified through subsequent histological analyses. Currently, the only minimally invasive method to obtain histological samples is through needle biopsies. Therefore, a combination of imaging and minimally invasive needle biopsies should be considered. Previous studies have shown that minimally invasive needle biopsies can have an overall diagnostic added value in combination with post-mortem imaging for the determination of the cause of death and major pathologic findings, although diagnostic accuracy for numerous specific diagnostic findings has yet to be thoroughly investigated [29, 30].

Based on previous studies, coronary thrombosis and central pulmonary embolism, for example, could alternatively be diagnosed using PMCT angiography [25, 26]. However, particular expertise and experience are needed for conducting PMCT angiography, which is the reason why this technique is currently conducted solely at a few forensic centers worldwide. In hospitals without access to such centers, conducting PMCT angiography is, at present, unlikely to be feasible in a non-forensic setting. For a wider-spread system of investigating deceased non-forensic hospital patients who died of natural causes of death, an approach of conducting combined unenhanced and widely accessible PMCT and PMMR may be a more feasible approach. However, from the authors’ point of view, the difficulty of accessing imaging equipment in non-forensic hospital settings is one of the main reasons why post-mortem imaging is currently not performed in clinical autopsy cases. In hospital settings, cadaver scans usually are not performed alongside routine patient procedures to avoid disturbing patients. Furthermore, experience has shown that not all radiology technicians want to have contact with deceased patients. As a result, cadaver scans are performed outside of regular operating hours, such as early in the morning, late in the evening, or at night. The resulting logistical challenges are sometimes so great that the scans are sometimes omitted altogether [29, 30]. Another reason for not performing post-mortem imaging in non-forensic hospital settings is presumably the lack of knowledge among clinical physicians and pathologists about the diagnostic capabilities of imaging [1–3]. For post-mortem imaging to be implemented in hospital settings, these logistical hurdles would first have to be overcome, and physicians would have to be further trained in this area. Also worth mentioning in this context are possible legal differences regarding forensic autopsy and clinical autopsy, which may influence the conduct of clinical post-mortem imaging. The study was conducted in Switzerland, where clinical autopsies and examinations of deceased persons require the explicit consent of the relatives. The public prosecutors, on the other hand, order forensic autopsies and post-mortem examinations of a body do not require consent [38]. The requirement of consent for clinical post-mortem imaging may therefore further complicate the process logistically. However, during the study, it became apparent that relatives who consented to a clinical autopsy generally also agreed to post-mortem imaging and would even have preferred this to autopsy. From this, one could conclude that the number of post-mortem examinations could possibly be increased through post-mortem imaging.

In previous studies, imaging was inferior to autopsy in determining the cause of death [14, 15, 20, 27, 28]. However, PMMR showed a higher overall diagnostic accuracy for determining the cause of death than PMCT in the present study. This is probably because natural causes of death such as myocardial infarction, coronary thrombosis, and neoplasms were relatively common in the study cases, for which PMMR is better suited than unenhanced PMCT. Forensic studies often include trauma cases, in which the cause of death can often be identified better with PMCT than with PMMR. However, Roberts et al. also found a higher cause of death sensitivity for PMMR in their study of forensic cases after certain forensic cases were not considered for the statistical calculations [14]. Overall, the results suggest that in clinical autopsy cases, a combined use of PMCT and PMMR promises greater diagnostic accuracy for the undirected search for a cause of death than just one method. However, for the directed search for a specific cause of death, one method may be sufficient. For example, it would be sufficient to perform just a PMMR when aiming to identify fatal myocardial infarction. A PMCT would also be sufficient for the confirmation of pneumonia or pericardial tamponade due to aortic dissection.

## Limitations

Although the present study has relatively high case numbers for adult clinical autopsy cases overall, certain pathological findings are nevertheless present in overall low numbers. Since some relevant pathological findings, such as myocarditis, are fundamentally rare, they could not be examined at all or only with very small case numbers. Further studies of this kind with higher case numbers, preferably also multicenter studies, should be carried out to determine the diagnostic accuracy for rare relevant findings.

In the case of PMMR in particular, the diagnostic accuracy for some pathological findings is likely to depend significantly on the sequence selected and the organ specifically targeted. The PMMR sequences selected for the study were predominantly standard sequences that were intended to cover the entire body or only particular organs such as the heart. It can be assumed that using specific MR sequences and focusing on individual organs, such as the liver, could result in a higher accuracy rate for specific diagnoses such as fatty liver disease.

Since bodies were cooled after death and the post-mortem interval (between death and imaging) was kept relatively short, no relevant putrefaction changes or putrefaction gas were generally observed. However, it can be assumed that in hospital settings where bodies are not cooled or when there is a longer post-mortem interval, putrefactive changes may occur, which may complicate or even prevent imaging diagnoses [15, 16, 19].

## Conclusion

Unenhanced PMCT and/or PMMR cannot serve as a general alternative to the classical clinical autopsy since a variety of relevant findings and specific diagnoses cannot be detected by either method. However, when the objective of a post-mortem examination is to confirm or investigate specific causes of death or a specific type of pathology, post-mortem imaging may offer a viable alternative to clinical autopsy. The combined use of PMCT and PMMR may slightly enhance diagnostic accuracy of some particular findings, but the combined use is not always necessary nor useful. Further studies, preferably multicenter with higher case numbers and combined with methods such as minimally invasive needle biopsy, should evaluate the diagnostic accuracy of relevant specific diagnoses that are beyond the scope of conventional unenhanced PMMR or PMCT.

## Supplementary information

Below is the link to the electronic supplementary material.

ESM 1  (XLSX 13.3 KB)

ESM 2 (XLSX 16.4 KB)

ESM 3 (XLSX 14.5 KB)

ESM 4 (XLSX 14.2 KB)

## Data Availability

Detailed datasets for the study comparison between autopsy and imaging diagnoses can be found in a repository via the following URL: 10.5281/zenodo.10006516.
